# Assessing the Validity and Reliability of the Russian Version of the Leading a Culture of Quality in Infection Prevention Scale among Nurses in Kazakhstan

**DOI:** 10.1155/2023/5309218

**Published:** 2023-09-07

**Authors:** Jonas Preposi Cruz, Paolo Colet, Joseph Almazan, Anargul Kuntuganova, Alma Syzdykova, Gaukhar Agazhayeva

**Affiliations:** ^1^Department of Medicine, School of Medicine, Nazarbayev University, Kerey and Zhanibek Khans St 5/1, Astana City 010000, Kazakhstan; ^2^Department of Biomedical Sciences, Nazarbayev University, Kerey and Zhanibek Khans St 5/1, Astana City 010000, Kazakhstan; ^3^Education Department, Corporate Fund University Medical Center, Astana City, Kazakhstan; ^4^Department of Epidemiologic Control, Corporate Fund University Medical Center, Astana City, Kazakhstan

## Abstract

**Background:**

Worldwide, hospitals are required to prioritize the culture of quality in infection prevention (CQIP) for patient safety. Assessing CQIP is crucial, but there is limited research, especially regarding nurses' perceptions. Insufficient information exists due to scarcity of validated instruments in local languages to measure CQIP internationally.

**Purpose:**

This study assessed the “Leading a Culture of Quality in Infection Prevention Scale” Russian version's (LCQ-IPS-R) validity and reliability to assess the CQIP of hospitals in Kazakhstan based on nurses' perceptions.

**Methods:**

This study utilized a methodological design and analyzed data from 204 nurses at the “National Research Center for Maternal and Child Health” in Astana City, Kazakhstan. The “cultural and linguistic adaptation” process involved a “forward-backward translation” technique. Content validity and construct validity were examined. Internal consistency reliability was explored for scale reliability.

**Results:**

The scale's mean item range was from 3.56 (SD = 1.22) to 4.40 (SD = 0.85; SD = 0.92). The corrected item-total correlation ranged from 0.321 to 0.707. Six experts rated the I-CVI from 0.83 to 1.00, with an S-CVI/Ave of 0.90. The principal component analysis with Varimax rotation produced four distinct components of the LCQ-IPS-R, explaining 69.8% of the total variance. The tests of correlation between the LCQ-IPS-R's four components revealed medium to large positive associations among the components (*r* = 0.25–0.55, *p* < 0.001). The computed *α* for the LCQ-IPS-R was 0.909 while *α* values from four subscales ranged from 0.809 to 0.921. *Conclusions/Implications for Practice*. This study provides evidence of the LCQ-IP-R's reliability and validity in evaluating Russian-speaking nurses' perception of their hospital's CQIP. These findings open the door for further research on CQIP in healthcare settings in Kazakhstan, Central Asia, and other Russian-speaking countries. The scale provides essential baseline information to design effective interventions for achieving hospitals' infection prevention objectives.

## 1. Introduction

Healthcare administrators and policymakers are continuously engaging in developing an infection prevention culture that is very extensive, convenient, and economical in healthcare settings. According to Cruz ([1]; p. 3), a “culture of quality in infection prevention” (CQIP) is defined as the “shared perception among healthcare professionals towards hospital infection prevention.” Hospitals worldwide are expected to have the highest level of CQIP to ensure patient safety and better patient outcomes. The World Health Organization [2] reported that routine infection prevention practices in Kazakhstan are well documented, including risk assessment for infectious diseases, patient safety, and establishing primary healthcare coordination centers staffed by healthcare professionals.

The roles of nurses are critical in ensuring a CQIP in hospitals [3]. They are expected to comply with infection prevention measures and to participate in interventions and activities that promote a culture of infection prevention [3]. They are also a good source of information when assessing the CQIP of hospitals. However, despite the important part of nurses in infection prevention in hospitals, nurses' compliance with infection prevention measures remains substandard [3–5]. Abed Alah et al. [6] reported that inconsistent infection prevention and control (IPC) interventions increased the chances for healthcare-associated infections (HAIs), leading to prolonged hospital stays. Hence, efforts should focus on nurses, who play a vital role in infection prevention, to prevent the detrimental effects of noncompliance to infection prevention practices on patient safety [4]. Establishing baseline data on the organizational culture of infection prevention could lead to better infection prevention management. It could enable nurses to improve their compliance with infection prevention policies and guidelines [7].

The significance of assessing the CQIP in hospitals cannot be overstated. However, there needs to be more information on this area of research in Kazakhstan, specifically on the perceptions of practicing nurses. The lack of a validated instrument in the country's language to measure the CQIP of a hospital is one of the main reasons for the lack of data from this country. A recent study conducted by Abed Alah et al. [6] has thoroughly investigated this tool which contributed to the understanding of how to assess the reliability and validity, assuring its usefulness and robustness in several research settings. Likewise, having a valid and reliable tool will be instrumental in accurately measuring the perceptions of healthcare workers about their hospital's infection prevention climate, which could prompt improvements and innovations in infection prevention practice within healthcare settings [4, 8]. By using a valid and reliable LCQ-IPS-R, researchers and healthcare administrators can effectively assess nurses' infection prevention efforts, recognize improvement areas, and implement strategies in preventing and reducing HAIs. Hence, it is crucial to have a validated and reliable version of a tool that can be used to assess the CQIP of hospitals in Russian-speaking countries to ensure that studies in this area can be done in those countries. Conducting validity and reliability test on LCQ-IPS-R is also key in ensuring that this scale's version is a robust and accurate measurement tool for assessing the culture of quality in infection prevention within healthcare organizations.

## 2. Background

Healthcare-associated infections (HAIs) remain a significant challenge among hospitals worldwide, posing significant risks to patient safety and outcomes [9]. According to “European Center for Disease Prevention and Control” ([10], para. 1), “healthcare-associated infections are infections acquired by patients during their stay in a hospital or another healthcare setting.” These preventable infections negatively impact the patient's “health, increasing their stay in the hospital and hospital costs, and causing considerable distress to these patients” ([10], para. 1). Some literature reported that HAIs increase the occurrence and undesirable complications. For example, before the pandemic, more than 4 million patients in Europe every year acquired HAIs, while approximately 37,000 died because of direct contact consequences [11]. In the United States, HAIs were listed as the fifth most common mortality causes in clinical settings [12]. In Africa, poor infection control practices in public hospitals pose a significant adverse public health challenge due to healthcare provider or patient-related factors.

In Kazakhstan, HAIs had increased, and the country's epidemiological situation is still a concern [13]. The COVID-19 pandemic contributed to many cross-infections between patients and nurses in the country, which posed a considerable challenge to healthcare institutions [14]. Due to this increase in HAI cases, the country's healthcare ministry has demonstrated its commitment to mitigating the impact of HAIs. The ministry also ensured access to essential healthcare services to accelerate improvement in health outcomes and respond better to its people's ever-changing needs and expectations [15]. The ministry promoted multidisciplinary teams in hospitals, supported by various health systems, to provide guidance in public health protection, one of which is IPC intervention.

IPC measures decrease the burden of unwanted hospital incidences and enhance the quality of health care in hospitals [9]. Accordingly, ensuring CQIP significantly impacts patient outcomes, including increased effectiveness of prevention strategies for hospital infections, promotion of effective communication, and encouraging individuals to learn from their mistakes [1, 16]. Hence, creating a CQIP in hospitals should be a joint priority among healthcare professionals, policymakers, administrators, and managers.

Nurses, who are primary care providers and have close contact with patients, are obligated to provide safe and effective nursing care and contribute to creating an infection-free environment [17]. Nursing has a crucial role in ensuring a safe patient care environment; thus, they must be competent in infection control skills. Moreover, they should also be competent in patient need prioritization, teamwork, and collaboration, which are essential in developing CQIP in hospitals [1]. Healthcare workers' lack of knowledge and competence in infection prevention often leads to failed CQIP [18]. Hence, assessing the hospital's CQIP as perceived by healthcare workers is critical to identify the areas that need to be intervened and develop interventions to ensure high IPC knowledge and competence.

The literature supports ways to improve knowledge and compliance on IPC among nurses. Alshammari et al. [7] found that IPC training seminars significantly influenced IPC compliance. Furthermore, attending “risk assessment seminar training, having sufficient personal protective equipment, and being completely aware of safety guidelines were related to better IPC practices” [5]. Data from this previous literature provide a valuable baseline understanding of nurses' knowledge and practices of IPC, which could be essential in understanding the CQIP of the healthcare setting.

Considering the relevance of CQIP to patient safety, it is vital to use psychometrically sound tools in measuring the CQIP. A validated tool for assessing a hospital's CQIP is the “Leading a Culture of Quality in Infection Prevention Scale” (LCQ-IPS) [19]. The LCQ-IPS is the most widely used tool to measure healthcare workers' perceptions about CQIP in a clinical health setting. It measures four dimensions of CQIP: “improvement orientation,” “psychological safety,” “supportive work environment,” and “prioritization of quality” [19]. To date, the psychometric properties of the LCQ-IPS have been tested in the US [19] and Saudi Arabia [1]. No Russian version of the tool is currently available, and no study has attempted to adapt and test the tool's validity and reliability among nurses in Kazakhstan. A valid and reliable Russian language version of the tool is necessary to assess the CQIP of hospitals in the country and other Russian-speaking countries. This situation will also hinder the conduct of cross-cultural comparisons on the CQIP between countries. Therefore, this study endeavored to produce a Russian version of the LCQ-IPS (LCQ-IPS-R) and test its psychometric properties among nurses in Kazakhstan.

## 3. Aim

This study assessed LCQ-IPS-R's validity and reliability to assess the CQIP of hospitals in Kazakhstan based on nurses' perceptions.

## 4. Research Questions

1. To what extent can the LCQ-IPS-R demonstrate content and construct validity when administered to Kazakh nurses?
2. What internal consistency reliability level does the LCQ-IPS-R and its subscales exhibited when administered to Kazakh nurses?

## 5. Method

### 5.1. Design

This study is a quantitative investigation utilizing a methodological design.

### 5.2. Samples and Setting

The data for this study were collected at the “National Research Center for Maternal and Child Health” (NRCMCH). The NRCMCH is a clinical research facility, one of the three medical centers under the “University Medical Center Corporate Fund” (UMC) in Astana City, Kazakhstan. There were approximately 950 nurses employed in the UMC hospitals, and invitations were extended to them to participate voluntarily. However, due to the nature of convenience sampling, only 204 nurses responded and voluntarily completed the questionnaire (response rate: 25.3%). However, since this is a validation study, the researchers used the “sample to scale item ratio” (1 : 10 ratio) in identifying the sample size, which is the commonly used method in identifying the adequate sample size when conducting factor analysis [20]. Since there are 19 items in the scale, the needed sample size was 190 nurses. Hence, the present sample size was more than adequate for the study [20]. The following were the inclusion criteria: (1) citizen of Kazakhstan, (2) staff nurse at NRCMCH, (3) with nursing or midwifery certificate, (4) reads and understands the Russian language, and (5) with at least six months working experience at the NRCMCH.

### 5.3. Instrument

The online survey contained questions to elicit data for the respondents' age, sex, marital status, education, and length of experience. The online survey also contains the LCQ-IPS-R, translated from the original English version by Pogorzelska-Maziarz et al. [19]. The “cultural and linguistic adaptation” process followed a “forward-backward translation” technique [21]. Two translators independently translated the scale from English to Russian language. Another translator synthesized the two translations to develop a single tentative Russian translation. Two translators separately back-translated the tentative Russian version to English. The tentative Russian version and the two back translations were presented to a panel of experts (on infection control and prevention) with six members. The panel examined the “semantic, idiomatic, experiential, and conceptual equivalence” between those versions. The panel also examined the tool's content validity following Polit and Beck's [22] recommendations.

The LCQ-IPS comprises 19 items that capture the central dimensions of a healthcare organization's climate of quality of IPC. The items of the scale were originally from the “Leading Culture of Quality” questionnaire created by the “Institute for Clinical Systems Improvement and Satisfaction Performance Research” in Minnesota to be used by healthcare organizations to assess their “quality-oriented climate.” Pogorzelska-Maziarz et al. [19] adapted the items and revised them to be focused on infection prevention. The scale can measure four aspects of the IPC climate of a hospital, including “psychological safety, prioritization of quality, supportive work environment, and improvement orientation.” The items are responded to using a 5-point Likert scale (1 = “strongly disagree” to 5 = “strongly agree”). Mean dimension scores are calculated after reverse scoring item number 16 as it is a negatively worded item. Higher mean scores indicate a better or more positive IPC climate of the hospital. The scale had been used to assess the nurses' [4] and nursing students' [1, 8] perceptions of their hospitals' IPC climate. The LCQ-IPS had good internal consistency (Cronbach's *α* = 0.926), criterion validity, and construct validity [19]. The copyright holder of the LCQ-IP permitted the use and translation of the tool through e-mail (M. Pogorzelska-Maziarz, personal communication, August 20, 2021).

### 5.4. Ethical Considerations

This report is part of a research protocol approved by the Institutional Research Ethics Committee of Nazarbayev University (Register Number: 448/24092021) and the Research Ethics Committee of UMC. The study strictly adhered to the guidelines set by these committees in the ethical conduct of research and the Declaration of Helsinki on ethical principles in researching human subjects. The IRB assessed the study protocol, research instruments, and ethical considerations to ensure compliance with ethical guidelines and regulations. The approval was obtained before initiating data collection. Information about the study (study's purpose, procedures, risks and benefits, and voluntary nature) and the rights (right to refuse and withdraw from participating) of the respondents was provided at the beginning of the online survey. The contact information of the principal investigator was provided on the same part of the survey in case the respondents have clarifications or questions about their study. Participation in the study was voluntary, and those who decided not to participate were free to ignore and leave the online survey. Those who volunteered to join signed an electronic informed consent form by clicking the “I agree” button at the end of the consent. No personal information that can identify the respondent was collected, and data were treated and reported aggregately. The researchers programmed Survey Monkey to collect strictly anonymous responses (i.e., no IP address, e-mail address, or name will be saved). Moreover, in the tool settings, nurses were allowed to submit their responses only once, preventing them from submitting multiple responses. A rigorous data cleaning procedure was also conducted to identify and remove any duplicated entries. Data were handled carefully, with aggregated and nonidentifiable results presented. By upholding this ethical consideration, the online survey was conducted with integrity, ensuring that the participant rights, privacy, and confidentiality were protected.

### 5.5. Data Collection

The researchers sought permission to conduct the study from the head of NRCMCH, outlining the purpose, objectives, and methodology of the study. The data for the study were collected from November 2021 to April 2022 using an online survey (Survey Monkey). The online survey's link, containing the study's information, electronic informed consent, and questionnaire, was forwarded to the nurses working in NRCMCH through e-mail and WhatsApp. A reminder message was sent to the potential respondents every two weeks to remind them about the survey. The collected data downloaded from Survey Monkey were securely stored and managed to ensure confidentiality and data integrity. The research team organized and documented the data in a structured format, making it ready for analysis.

### 5.6. Data Analysis

Analyses were conducted using SPSS version 22.0. Descriptive analyses were performed to analyze the demographic variables in the study. The “item-level” (I-CVI) and “scale-level” content validity indices (S-CVI/Ave) were computed from the ratings given by a panel of six experts. The acceptable I-CVIs and S-CVI/Ave for a six-member panel of experts are ≥0.78 and ≥0.90, respectively [22]. Item-to-total correlation (ITC) was analyzed to support the scale's internal structure validity. Items with ITC values less than 0.30 and more than 0.80 and items that will cause ≥10% increase in the scale's “Cronbach's alpha score when removed” were dropped from the scale. For construct validity, PCA with Varimax rotation was carried out. The “sampling adequacy” and the “appropriateness of the factor model” were determined by calculating the “Kaiser–Meyer–Olkin index” (KMO value ≥ 0.60) and “Bartlett's test of sphericity” (*p* < 0.05), respectively. Extraction of factors will consider an eigenvalue >1 and factor loading of >0.40 [23]. For reliability, internal consistency reliability was determined. Cronbach's alpha (*α* ≥ 0.70) was calculated for the scale's reliability [24].

## 6. Results

The nurses' age ranged from 20 to 60 years (*M* = 34.63; SD = 10.73) years. Less than 10% of the respondents were males, and more than half of the sample was married (56.9%). Most of the respondents had finished a certificate in nursing or midwifery in a nursing college (56.9%). The year of experience as a nurse ranged from 0.80 to 40 years (*M* = 12.33; SD = 10.12) (Table 1).

### 6.1. Item Analysis on the LCQ-IPS Russian Version

The results of the item analysis conducted on the LCQ-IPS-R are summarized in Table 2. The mean range of the scale's items was from 3.56 (SD = 1.22) to 4.40 (SD = 0.85; SD = 0.92). The corrected ITC ranged from 0.321 (item 16) to 0.707 (item 8). Cronbach's *α* values “if the item is deleted” ranged from 0.901 (items 5 and 13) to 0.910 (items 17 and 18).

### 6.2. Validity of the LCQ-IPS Russian Version

The six experts rated the I-CVI from 0.83 to 1.00, with an S-CVI/Ave of 0.90. All the items were subjected to the PCA. The KMO value was 0.87, and Bartlett's test of sphericity was significant (*p* < 0.001); thus, the sample was adequate to continue with the PCA. The PCA with Varimax rotation produced four distinct components of the LCQ-IPS-R with an eigenvalue greater than 1 (range = 1.23 to 7.80). The overall explained variance of the four components was 69.8%. Seven of the 19 items were loaded in component 1 (factor loading range = 0.724–0.862) with the highest explained variance (41.0%). Component 2 explained 14.3% of the variance with seven items loaded (factor loading range = 0.482 to 0.795). For component 3, four items were loaded on it with factor loading from 0.528 to 0.868 and an explained variance of 8.0%. Three items were loaded in component four (factor loading range = 0.687 to 0.878), contributing to 6.5% of the variance explained. However, items 13 and 14 were cross-loaded in components 2 and 3. The researchers decided to retain both items in component 3. Therefore, the final model comprises the following: component 1, “psychological safety” (7 items); component 2, “prioritization of quality” (5 items); component 3, “supportive work environment” (4 items); and component 4, “improvement orientation” (3 items) (see Table 3).

The tests of correlation between the four components of the LCQ-IPS-R revealed medium to large positive associations among the components (*r* = 0.25–0.55, *p* < 0.001) (see Table 4).

### 6.3. Reliability of the LCQ-IPS Russian Version

The computed Cronbach's alpha for the LCQ-IPS-R was 0.909. For the subscales, Cronbach's alpha values of 0.921, 0.855, 0.813, and 0.809 were computed for “Psychological safety,” “Prioritization of quality,” “Supportive work environment,” and “Improvement orientation,” respectively (see Table 5).

## 7. Discussion

This study showed evidence of the validity and reliability of the LCQ-IPS-R in assessing the CQIP of hospitals in Kazakhstan as perceived by nurses. When evaluating the various aspects of the hospital's CQIP from a nurse's standpoint, it is vital to use a valid and reliable measurement scale. This psychometric assessment is the first to test the suitability of LCQ-IPS in the Kazakhstan context and Russian language.

The result of the content validity test revealed that the IPC experts evaluated all the LCQ-IPS-R items as relevant or highly relevant in assessing the CQIP of hospitals in Kazakhstan context and language. The LCQ-IPS-R had undergone a rigorous translation process using the “forward-backward translation method” from the original language of the scale (English) to Russian. The excellent content validity indices of the Russian version ascertained its equivalency in both linguistic and cultural contexts with the original version [25]. This excellent content validity exhibited that the translated items effectively capture the content and the meaning of the original tool [19]. Thus, the Russian version is parallel with its intended construct and maintains its validity when applied to Russian-speaking nurses in Kazakhstan. Previous studies reporting content validity of other scale versions also reported similar excellent content validity indices in various countries [1, 3, 19].

A descriptive analysis of the scale items was performed. The analysis reflected that “the healthcare-associated infection prevention goals and strategic plan of our organization are clear and well communicated” and “I have a clear understanding of the organization's mission, vision, and values” received the highest item mean. These high mean scores demonstrate that nurses clearly understood their hospital's goals and strategic plan related to HAI prevention goals, as well as their hospital's mission, vision, and values. This finding further suggests that the healthcare organization was successful in communicating with clarity regarding its infection prevention goals, strategic plan, and core values among its healthcare staff [16]. This result is worth noting since the health organization's values, goals, and strategic plan emphasizing the IPC climate are critical in promoting patient safety culture [6]. The nurses' knowledge and understanding of the hospital's mission, vision, and values could help them properly align their actions towards achieving quality and patient safety improvement, including reducing infection risk and protecting the patients and other healthcare professionals (Alquwez et al., 2018). Additionally, ITCs were used to support the internal construct validity of the tool. The result showed that all ITCs were between 0.30 and 0.80, which implies acceptable ITC values based on the definition of Nunnally and Bernstein [24]. This also means that the items are reasonably connected to the overall construct and contribute significantly to its measurement [23]. The “Cronbach's alpha if the item is deleted” values also showed that none of the items, when deleted, will cause a substantial increase in the scale's overall Cronbach's alpha. The findings indicate that all the items are relevant to the totality of the scale. Thus, they were entered into the PCA.

The tool manifested a suitable construct validity, as shown by the 4-factor solution of the PCA explaining 69.8% of the overall scale's variance. This means that the four distinct factors in the scale contributed about 69.8% of the total variance in the nurses' perceptions of their hospital's CQIP. This overall explained variance of the four factors in the LCQ-IPS-R is indicative that the tool had a good construct validity [26]. This explained variance was higher than the explained variance of the English version (58.8%) but lower than that of the Arabic version (70.7%) [1]. Similar to the LCQ-IPS, the subscales were labeled “Psychological safety,” “Prioritization of quality,” “Supportive work environment,” and “Improvement orientation” [19]. Our findings also confirm the previous results reported in the English and Arabic versions of the scale that “Psychological safety” (41.0%) has the most substantial contribution to the variance of CQIP, followed by “Prioritization of quality,” “Supportive work environment,” and “Improvement orientation” [1, 19].

However, items 13 and 14 were loaded on components 2 and 3. According to Al-Dwaikat et al. [27], if an item is loaded into two different domains, these items can be removed as it creates confusion in labeling all factors that share similar variables. However, Nunnally and Bernstein [24] argued that if items are loaded in at least two factors, they can be kept in the factor they had strong association. Item 13 states that “senior leadership here has created an environment that enables changes to be made,” and item 14 states “where I work, people are held accountable for the results of their work.” Both items were more related to the nurses' working environment than the concept of prioritizing quality. Also, items 13 and 14 were heavily loaded more on the “Supportive work environment” factor than in “Prioritization of quality.” A supportive nursing leadership that promotes accountability is a characteristic of a positive work environment [3, 28]. A systematic review by Wei et al. [29] also reported that nurses' safe and positive work environment, empowerment, autonomy, positive relationships with other team members, and organizational support significantly ensure IPC in hospitals. Therefore, in this study, we decided to retain both items in factor 3.

Factor 1, “Psychological safety,” constitutes seven items. This factor is composed of items describing a hospital that promotes the free exchange of ideas about IPC and patient safety. It also includes issues and challenges related to these aspects in the hospital. This implies that the nurses value a hospital climate that promotes openness to these issues. They believed that having an organizational culture that allows a free exchange of ideas impacts the attainment of IPC and patient safety in the hospital. The data collection for this study was conducted between November 2021 to April 2022, when significant changes happened in Kazakhstan's healthcare work environment and practices. Several measures were carried out to mitigate the spread of COVID-19 infection in workplaces [15]. Smith et al. [30] explained that appropriate infection control measure implementation in the clinical setting might positively affect healthcare professionals, such as reducing anxiety and worries about contact/disease transmission while performing patient care. In a systematic review conducted by Lawrence and Kinnear [31], psychological safety promotes teamwork, open communication, and continuous improvement culture. When individuals feel safe psychologically, they are more likely to share their valuable insights, report errors or near misses, and actively participate in ward decision-making processes. Thus, psychological safety is crucial in healthcare facilities to ensure the effective implementation of IPC policies and guidelines and the attainment of patient safety goals.

The second factor, “Prioritization of quality,” has five items. The items in this factor talk about the clarity of the hospital's IPC goals and plans, assessment of IPC efforts and communication of findings, effective communication regarding IPC, and the prioritization of IPC in the hospital. Hence, prioritizing the quality of IPC in hospitals during those times is essential to deliver safe and effective nursing care. Tumala et al. [8] reiterated that to ensure the best IPC practices in patient care, a clear technical guideline on IPC according to available resources and clear IPC goals and plans should be present. The “Ministry of Healthcare of the Republic of Kazakhstan” [32] mandated that all health centers, hospitals, and health clinics prioritize their IPC plan across all departments/areas to reduce the risk of infection and guarantee patient safety. Therefore, by prioritizing quality IPC, the country's healthcare system can enhance patient safety, improve healthcare outcomes, and reduce the HAI burden.

Four items constitute factor 3, “Supportive work environment.” These items tackle leadership support to IPC, accountability of healthcare workers, and the effects of high workload and inadequate time on IPC practices. This factor generates an insight that nurses' working environment influences the nurses' provision of efficient nursing service and effective IPC initiatives [33, 34]. During the pandemic, nurses rendered most of their time to patient care, often facing a higher patient load that demands attention [35]. As this factor shows, the time and workload of nurses are crucial in determining if they can effectively practice IPC. According to Park and Kim [36], a nonsupportive work environment is associated with high burnout, higher turnover intention, and lower job satisfaction. Hence, the work environment is crucial to creating safe health care and reducing infection risks in a clinical setting.

Finally, three items made up factor 4, “Improvement orientation.” This factor includes the following items: “I can think of examples when problems with patient infections have led to changes in our procedures or equipment,” “I know of one or more healthcare-associated infection prevention initiatives going on within our organization this year,” and “I have a clear understanding of the organization's mission, vision, and values.” This factor highlights the alignment of IPC goals and the healthcare organization's “mission, vision, and values.” Also, a healthcare organization that is serious about achieving IPC goals should have clear policies, guidelines, and plans in achieving its IPC goals. According to [9], regular assessments and monitoring practices and timely feedback for healthcare professionals are necessary to achieve continuous improvement orientation on infection control.

The scale's overall Cronbach's*α* alpha is 0.909, with a range of 0.809 to 0.921 for its four subscales. According to Nunnally and Bernstein [24], a criterion of 0.70 or above indicates acceptable internal consistency. It suggests that the scale's items are highly correlated with each other and holistically evaluate the intended construct in a consistent and reliable manner. This result is higher than that of other previous studies, which reported similar good internal consistency reliability using the same tool. For example, Tumala et al. [8] conducted a survey using the Arabic version among 829 Saudi nursing students in six Saudi universities, which found Cronbach's alpha of 0.89. Meanwhile, in the study by Colet et al. [4] among nurses in Riyadh, Saudi Arabia, Cronbach's *α* was 0.87, signifying good internal consistency. In addition, it is crucial to note that Cronbach's alpha coefficients are affected by the item number on an instrument measurement [37]. Thus, the LCQ-IPS-R exhibits excellent internal consistency.

### 7.1. Limitations

A few limitations need consideration when interpreting the study's results. The LCQ-IPS-R's validity was supported by “content validity” and “construct validity” through PCA. While these tests provided evidence for the tool's acceptable validity, other validity tests should be performed in the LCQ-IPS-R in the future. Also, the tool's structure was supported only by PCA. A CFA should be done to support LCQ-IPS-R's four-factor structure. The CFA omission might impact the validation process robustness and could influence the interpretation of the results. Therefore, further studies are warranted for CFA incorporation in enhancing the validity assessment and ensure a more comprehensive adapted scale validation. Also, the reliability of the tool was supported only by “internal consistency.” Other reliability measures, such as stability reliability, can be done in the future to strengthen the evidence of the reliability of this version.

## 8. Conclusion

This investigation substantially contributes to the literature on the IPC of hospitals worldwide and in Kazakhstan by measuring the LCQ-IPS-R's psychometric properties. This research provides evidence of the LCQ-IP-R's validity and reliability in assessing the Russian-speaking nurses' perception of their hospital's CQIP. Specifically, the LCQ-IP-R's validity was supported by excellent “content and construct validity.” The PCA affirmed the four subscales of the tool similar to the original versions, namely, “Psychological safety, Prioritization of quality, Supportive work environment, and Improvement orientation.” The LCQ-IP-R and its four subscales had high internal consistency reliability. Therefore, the LCQ-IP-R can provide a valid and reliable assessment of the CQIP in hospitals in Kazakhstan and other Russian-speaking countries from clinical nurses' perceptions. This scale offers the opportunity to derive baseline information enabling the design and implementation of effective interventions to ensure the attainment of IPC goals of hospitals.

### 8.1. Implications

The LCQ-IP-R was used for the first time among Kazakhstan's nurses, supporting the scale's validity and reliability. This result could pave the way for more research regarding CQIP of healthcare settings to be conducted in Kazakhstan, Central Asia, and other Russian-speaking countries. Also, the results are of great significance to the nursing profession as it established an effective measurement tool of CQIP, which can provide information for designing IPC policies and guidelines and planning IPC initiatives to ensure patient safety in clinical settings. Hospital administrators could use this tool to conduct continuous and regular assessments of the IPC situations in their hospitals. These assessments can improve the hospital's psychological safety, prioritize IPC, create a supportive working environment, and ensure quality improvement in IPC. Likewise, head nurses, managers, and nursing directors could actively promote CQIP by ensuring adequate resources for IPC measures using the baseline information gathered through the tool used and may stimulate the continuous improvement of IPC measures.

## Figures and Tables

**Table 1:** Demographic characteristics of the respondents (*n* = 204).

Variable	Mean (SD)	Range
Age	34.63 (10.73)	20–60
Years of experience	12.33 (10.12)	0.80–40

	*n*	%

Gender		
Male	20	9.8
Female	184	90.2
Marital status		
Single	68	33.3
Married	116	56.9
Separated/divorced/widow/er	20	9.8
Highest educational attainment		
Certificate in nursing or midwifery (nursing college)	116	56.9
Certificate in nursing or midwifery (higher medical college)	15	7.4
Feldsher	12	5.9
Baccalaureate in nursing (applied bachelor)	15	7.4
Baccalaureate in nursing (academic bachelor)	41	20.1
Graduate program (master or doctorate)	5	2.5

**Table 2:** Item mean, corrected item-total correlations, and Cronbach's *α* if item is deleted for the Leading a Culture of Quality in Infection Prevention Scale Russian version (*n* = 204).

No.	Item	Mean	SD	Corrected item-totalcorrelation	Cronbach's *α*if item is deleted
1	The climate in the organization promotes the free exchange of ideas	4.22	0.97	0.545	0.905
2	Staff will freely speak up if they see something that may improve patient care or affect patient safety	4.35	0.92	0.643	0.903
3	I feel free to express my opinion without worrying about the outcome	4.17	1.02	0.650	0.902
4	In general, people in our organization treat each other with respect	4.22	1.02	0.566	0.905
5	People in this organization are comfortable checking with each other if they have questions about the right way to do something	4.27	0.96	0.695	0.901
6	The people in this organization value others' unique skills and talents	4.21	1.05	0.603	0.904
7	Members of this organization are able to bring up problems and tough issues	4.13	1.03	0.636	0.903
8	The healthcare-associated infection prevention goals and strategic plan of our organization are clear and well communicated	4.40	0.85	0.707	0.902
9	Results of our infection prevention efforts are measured and communicated regularly to staff	4.33	0.80	0.626	0.904
10	There is a good information flow among departments to provide high-quality patient safety and care	4.29	0.84	0.658	0.903
11	People here feel a sense of urgency about preventing healthcare-associated infections	4.22	0.98	0.565	0.905
12	Employees are encouraged to become involved in infection prevention	3.96	1.10	0.551	0.905
13	Senior leadership here has created an environment that enables changes to be made	4.00	1.06	0.691	0.901
14	Where I work, people are held accountable for the results of their work	4.35	0.97	0.667	0.902
15	The quality of work suffers because of the amount of work staff are expected to do	3.67	1.25	0.453	0.909
16	Most people in this organization are so busy that they have very little time to devote to infection prevention efforts	3.56	1.22	0.321	0.913
17	I can think of examples when problems with patient infections have led to changes in our procedures or equipment	3.87	1.09	0.377	0.910
18	I know of one or more healthcare-associated infection prevention initiatives going on within our organization this year	3.94	1.02	0.376	0.910
19	I have a clear understanding of the organization's mission, vision, and values	4.40	0.92	0.529	0.906

**Table 3:** Results of the principal component analysis for the Leading a Culture of Quality in Infection Prevention Scale Russian version (*n* = 204).

Item	Factor 1 Psychological safety	Factor 2 Prioritization of quality	Factor 3 Supportive work environment	Factor 4 Improvement orientation
Q5	0.862			
Q4	0.833			
Q3	0.819			
Q2	0.786			
Q6	0.770			
Q7	0.733			
Q1	0.724			
Q9		0.795		
Q8		0.764		
Q10		0.735		
Q11		0.687		
Q12		0.635		
Q16			0.868	
Q15			0.831	
Q13		0.448	0.579	
Q14		0.482	0.528	
Q18				0.878
Q17				0.865
Q19				0.687
Eigenvalue	7.80	2.71	1.52	1.23
Variance explained (%)	41.0%	14.3%	8.0%	6.5%
Cumulative variance explained (%)	41.0%	55.3%	63.3%	69.8%

**Table 4:** Correlation between the three factors of the Leading a Culture of Quality in Infection Prevention Scale Russian version (*n* = 204).

Factors	Psychological safety	Prioritization of quality	Supportive work environment
Prioritization of quality	0.55 (<0.001^*∗∗∗*^)		
Supportive work environment	0.34 (<0.001^*∗∗∗*^)	0.55 (<0.001^*∗∗∗*^)	
Improvement orientation	0.25 (<0.001^*∗∗∗*^)	0.37 (<0.001^*∗∗∗*^)	0.41 (<0.001^*∗∗∗*^)

*Note*. ^*∗∗∗*^Significant at 0.001 level.

**Table 5:** Internal consistency reliability of the Leading a Culture of Quality in Infection Prevention Scale Russian version (*n* = 204).

Variable	Cronbach's alpha
Psychological safety	0.921
Prioritization of quality	0.855
Supportive work environment	0.813
Improvement orientation	0.809
Leading a Culture of Quality in Infection Prevention Scale Russian version	0.909

## Data Availability

The data used to support the findings of this study have not been made available because participants were assured of their privacy and confidentiality.
